# Light stimulation on tenocytes: A systematic review of in vitro studies

**DOI:** 10.1097/j.pbj.0000000000000176

**Published:** 2022-09-09

**Authors:** Mariana Rodrigues da Silva, Renato Andrade, Fatima S. Cardoso, Sofia Oliveira, Susana O. Catarino, Óscar Carvalho, Filipe S. Silva, João Espregueira-Mendes, Paulo Flores

**Affiliations:** a Center for MicroElectroMechanical Systems (CMEMS-UMINHO), University of Minho, Azurém Campus, Guimarães, Portugal,; b Clínica Espregueira - FIFA Medical Centre of Excellence, Porto, Portugal,; c Dom Henrique Research Centre, Porto, Portugal,; d Porto Biomechanics Laboratory (LABIOMEP), Faculty of Sports, University of Porto, Porto, Portugal,; e ICVS/3B’s-PT Government Associate Laboratory, Braga/Guimarães, Portugal,; f 3B’s Research Group-Biomaterials, Biodegradables and Biomimetics, Headquarters of the European Institute of Excellence on Tissue Engineering and Regenerative Medicine, University of Minho, AvePark, Parque de Ciência e Tecnologia, Zona Industria da Gandra, Barco, Guimaräes, Portugal,; g School of Medicine, University of Minho, Braga, Portugal.

**Keywords:** light stimulation, Photobiomodulation Therapy, tendinopathy, tendon, tendon fibroblast, tenocyte

## Abstract

The purpose of this systematic review is to analyze the methodologies, utilized stimulation parameters, and the main cellular outcomes obtained by in vitro studies that apply a light source on tenocyte cultures.

**Methods:**

The PubMed, Scopus, and Web of Science databases were searched up to December 9, 2019 for in vitro studies that used light sources on tenocyte cultures. A 13-item checklist was used to assess methodological quality of the studies and the risk of bias was assessed using the Risk of Bias Assessment tool for Non-randomized Studies tool.

**Results:**

Six studies were included. Tenocytes from the Achilles tendon were used by 83.3% of the studies, with 16.7% utilizing the deep digital flexor tendon, with cells in passage 2 to 5. Four studies used lasers and the other 2 used light-emitting diode or intense pulsed light, in wavelengths ranges from 530 to 1100 nm. The application of light to tenocytes resulted in positive effects reported by all studies, including an increase in cell proliferation and migration, and higher protein and gene expression of tendon biomarkers. Studies presented a lack of standardization on reporting light stimulation parameters and experimental methodologies, leading to low methodological quality. There was a high risk of selection, performance, detection, and reporting bias.

**Conclusions:**

All studies showed positive effects after light stimulation on tenocytes, regardless of the light source used. However, the lack of standardized data on light stimulation parameters, experimental setup, and the studies’ main limitations hindered representative conclusions and comparisons amongst studies’ main outcomes.

## Introduction

Tendons are composed by tenocytes and an extracellular matrix constituted by glycoproteins, proteoglycans and water, embedding collagen and elastin fibers.^1^ As a macrostructure, tendons connect muscles to bones, transmitting muscle force to the skeleton, which enables joint motion.^1,2^ Due to their function, tendons are subjected to mechanical loading during daily activities, and, therefore, load is fundamental for their homeo-stasis, being beneficial for tendon remodeling.^3,4^

Overload and/or repetitive microtrauma within physiological limits may cause tendon injury, leading to pain that significantly impairs patient’s activities.^5,6^ Tendon-related pain is referred to as tendinopathy, being a musculoskeletal disorder (MSD).^6,7^ Each year, 33 million MSD are reported in the United States, 50% of which involving tendons and ligaments, costing approximately $30 billion.^8,9^ Tendinopathy may arise due to a variety of factors, and thus there is an abundance of time-consuming non-operative treatments aiming at reducing symptoms and enhancing tendon healing.^10^ These include non-steroidal anti-inflammatory drugs, therapeutic ultrasound, electrotherapy, heat/cold therapy, therapeutic exercise, and manual therapy.^10−12^ It remains unclear which of these treatments is more effective,^10^ and recurrences are common, particularly if patients return to their previous level of activity.

Alternative treatments to MSD have been emerging, some of which being based on light stimulation. There is a variety of designations for these treatments, such as Low Level Laser (or Light) Therapy, Low Intensity Laser Therapy, and Low Power Laser Therapy. There is clearly a lack of consistency and consensus on reporting the terminology associated with these therapies, highlighting the need for a consensual nomenclature. The term Photobiomodulation Therapy (PBT) is now used to describe these therapies, being defined as a light therapy, based on a non-thermal process, utilizing non-ionizing light sources (lasers, light-emitting diodes [LEDs], and broad-band light) in the visible and infrared spectrum.^10,13^ The mechanism of action of PBT is thought to be the absorption of red and near-infrared radiation by cytochrome c oxidase that is present in the cellular mitochondria. This mechanism results in the activation of signaling pathways, which in turn activates transcription factors, altering cellular metabolism and function.^14,15^ The latest investment in the research area of PBT is supported by experimental evidence of its biological effects on tendon injuries, comprising higher adenosine triphosphate (ATP) production, improved cellular and metabolic function, higher cell proliferation, higher protein synthesis, reduction in inflammation, upregulation of collagen (protein expression), and angiogenesis.^10^

In vitro,^16,17^ in vivo,^18,19^ and clinical studies^20−22^ have been and continue to be carried out to assess the efficacy of PBT as a treatment of tendon injuries. However, its efficacy is dependent on the correctness of the applied stimulation parameters.^15^ Despite the extensive investigation on this research topic, it is still lacking a standardized reporting methodology that pinpoints differences amongst studies on the in vitro application of PBT to tenocytes. A systematization and a detailed analysis of this data is warranted and may facilitate the definition of guidelines for future research. The purpose of this systematic review is to summarize the evidence of in vitro studies regarding the methodologies, used stimulation parameters and main cellular outcomes after applying PBT on tenocytes. Our goal is to provide a standardized method of reporting for future studies and to outline the current state-of-the-art knowledge of PBT as a treatment of tendon injuries.

## Methods

The present systematic review of literature was conducted according to the Preferred Reporting Items for Systematic Reviews and Meta-Analyses (PRISMA) guidelines.^23^

### Search strategy

A comprehensive electronic database search was carried out on MEDLINE/PubMed, Scopus, and Web of Science databases to identify studies that analyze the effect of a light source (for instance, laser or LED) on tenocytes. The searches were performed from database inception up to December 9, 2019. The search strategy was established by the use of AND/OR Boolean operators and combining the following keywords: tenocyte, “tendon cell”, tendon, “tendon fibroblast”, “in vitro”, “cell culture”, “cell therapy”, photobiomodulation, LED, phototherapy, and laser. These keywords were introduced with the field tag [All Fields] within the database searches, except for Scopus database, which was searched by [Title/Abstract/Keywords]. An example of the search is shown in Appendix A available in Supplementary Digital Content, http://links.lww.com/PBJ/A11.

### Study selection

All records were extracted to an Excel file (Microsoft^®^ Office version 16.16.18) and duplicates were removed by software filter and then manually verified. The reference lists of relevant studies resulting from the database search were analyzed to identify other studies that could potentially be included. Two authors (MS and RA) screened all titles and abstracts and identified relevant studies that were retrieved for full-text analysis. We included studies analyzing the effect of a light source (for instance, laser or LED) on tendon cells. Studies were excluded if they (1) did not use in vitro experimental procedures; (2) did not utilize tendon cells, tenocytes, or tendon fibroblasts; (3) did not employ a light source; (4) analyzed the effect of a chemical compound (for instance, hyaluronic acid) on cells; (5) included the use of scaffolds or other tissue-engineered constructs; and (6) utilized computational models. Other reviews or meta-analyses, case studies, and/or expert opinions, as well as studies not written in the English language were also excluded.

### Data collection and extraction

Customized data extraction tables were developed to extract key details from each included study considering: (1) species and number of samples utilized; (2) tendon from where the cells were derived; (3) light source stimulation parameters, including, for instance, wavelength, energy density, and optical power; (4) experimental conditions; (5) experimental techniques for analysis of results; and (6) main findings of the study. These characteristics were chosen to provide an overview of the methodologies each study employed in the performed analyses and the results and conclusions obtained. When only partial information was described in the original study, references and authors’ previous works were analyzed to provide comparable data across selected studies.

### Methodological quality

A checklist was developed to assess the methodological quality of the studies included in this systematic review. The checklist was based on a previous scale^24^ and adapted to the scope of this systematic review. Each question from the checklist was scored as 0 (no information), 1 (limited details), or 2 (satisfying description). The 13-item quality checklist used in this review was: Q1: Are the research objectives clearly stated? Q2: Is the number of samples and species from where tendon cells were derived adequately described? Q3: Is the tendon from where cells were derived adequately described? Q4: Are the light stimulation parameters clearly defined? Q5: Is the experimental setup, utilized equipment, and evaluation procedures clearly defined? Q6: Are the direct results easily interpretable? Q7: Are the main outcomes clearly stated and supported by the results? Q8: Are the limitations of the study clearly described? Q9: Are key findings compared with other literature? Q10: Is the frequency of exposure reported? Q11: Is the ethical committee approval reported? Q12: Are the control and exposure groups clearly defined? and Q13: Is the statistical analysis clearly described? The overall score (%) of each study was calculated as the sum of the classifications attributed in all questions divided by the sum of the maximum classifications of all questions.

### Risk of bias assessment

We appraised the risk of bias of the selected studies using the Risk of Bias Assessment tool for Non-randomized Studies tool. The Risk of Bias Assessment tool for Non-randomized Studies is a validated tool to assess the risk of bias of non-randomized studies. It appraises 6 domains including the selection of participants, confounding variables, measurement of exposure, blinding of outcome assessment, incomplete outcome data, and selective outcome reporting. We adapted to criteria to the context of our systematic review (ie, in vitro studies) and added 2 additional domains — planning and implementation of interventions, and funding bias — which were relevant for our systematic review (Table 1).^25^ The risk of bias appraisal was performed by 5 authors (MS, RA, SC, SO, and FC).

**Table 1:** Domains and their description for the appraisal of the risk of bias using the Risk of Bias Assessment tool for Non-randomized Studies (RoBANS) tool.

Domain	Description
Selection of tendon specimens and cells	**Selection bias** caused by inadequate selection of tendon specimens from where primary cell lines are established and by inadequate selection of cells.
	Collection of tendon specimens should be performed in the same conditions^*^ and allocation (cells and tissue source) should be randomized. Tendon cells should be isolated from more than 1 animal. Control and intervention groups should be clearly defined. Tenocytes should be confirmed positive for specific cell surface markers, such as CD44, CD90, and CD105.^25^
Confounding variables	**Selection bias** caused by inadequate confirmation and consideration of confounding variables.
	Studies should comprise the same animal species (if animal studies), same tendon type (eg, Achilles or DDFT), same cell viability and count/density, and same number of cell passages. Studies should implement the same tendon specimen isolation protocol and the same protocol for establishing the primary cell culture(s). The same experimental conditions should be guaranteed for both the control and exposure groups (eg, humidity, CO_2_, and temperature conditions). The volume of culture medium in all groups should be the same.
Planning and implementation of interventions	Performance bias caused by inadequate planning and implementation of interventions.
	The samples should be prepared by the same operator. Calibration and control of light stimulation parameters should be performed prior to, during and/or after interventions. During the stimulation procedures, the radiation scattering between the wells of the same culture plate must be considered (eg, use of black culture plates). Light stimulation procedures/methodologies should be clearly explained (eg, distance and angle of light source to cell culture and time of exposure). The light source (eg, laser, LED) and stimulation parameters (eg, optical power, wavelength, and number of actuators) should be clearly described for all experimental groups. During interventions, temperature should be controlled (PBT should not induce a temperature increase in tissues or cells^26−28^). Studies should clearly report the number of light stimulations applied in each group. Experiments should be replicated at least 3 independent times.
Exposure measurement	Blinding of personnel or testing source (cells) is not possible. In these interventions the parameters (frequency, intensity, and distance) are pre-determined, the personnel who applies the intervention (PBT) cannot change the intervention or affect the outcomes. Thus, we did not judge performance bias related to blinding of personnel or testing source.
	**Performance bias** caused by inadequate measurement of exposure.
	Assessment of outcomes should be performed according to acceptable or well-established techniques for the specific outcome that studies are assessing. Semi-quantitative and qualitative analysis should be performed by 2 independent observers to ascertain intra-operator reliability.
Blinding outcome assessment	**Detection bias** caused by inadequate blinding of outcome assessment.
	Outcome assessor and/or data analysist not blinded to group (ie, intervention vs control). For quantitative analyses, the blinding of outcome assessor and/or data analysist was not considered necessary. Otherwise (semi-quantitative and qualitative analyses), blinding is required.
Incomplete outcome data	**Attrition bias** caused by inadequate handling of incomplete data outcome.
	Missing data in >5% of outcome variables.
Selective outcome reporting	**Reporting bias** caused by selective outcome reporting.
	Based on reporting of the collected/assessed outcomes and multiple subgroup analyses.
Funding bias	**Funding bias** caused due to financial sponsoring or conflict of interest.
	Conflict of interest from study authors and/or sponsoring of industry.

DDFT = Deep digital flexor tendon, LED = light-emitting diode, PBT = Photobiomodulation Therapy.

* Same species, same anatomical location, and similar weight and age of the animal.

## Results

### Search strategy

**Figure 1:**
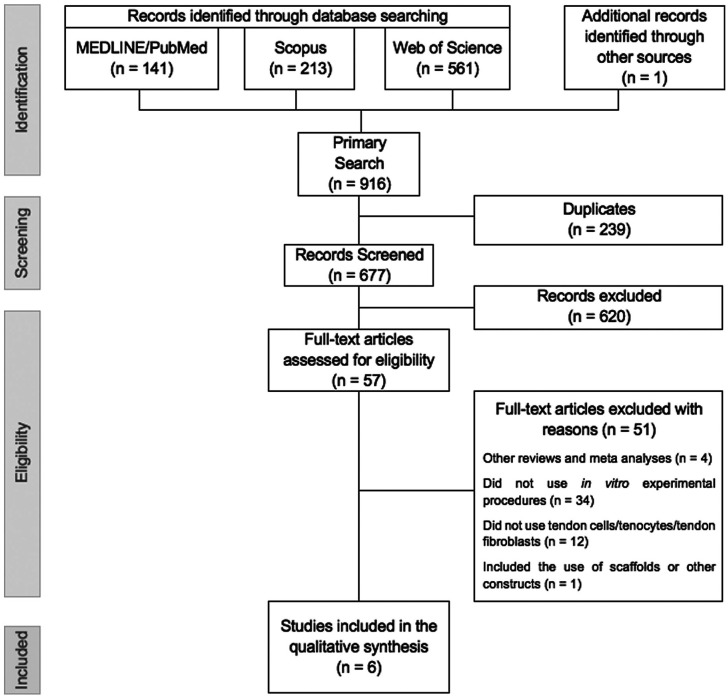
Flowchart of the search strategy conducted In this systematic review.

The database and manual searches provided a total of 916 results. After duplicates were removed, we screened 677 titles and abstracts, resulting in 57 studies that were retrieved for full-text analysis. A total of 6 studies met the eligibility criteria and were included in this systematic review (Fig. 1).

### Methodological quality assessment

The methodological quality scores ranged from 69.2% to 80.8%, with a mean value of 76.9% (Appendix B available in Supplementary Digital Content, http://links.lww.com/PBJ/A11). The lowest scores were found for Q4, Q5, and Q8, indicating the lack of methodological information regarding detailed reporting of light stimulation parameters, explanation of the experimental setup, utilized equipment and evaluation procedures, and limitations of the studies, respectively. Three studies^16,26,27^ did not report any limitations from the research (scored 0). All studies clearly specified the aim of the developed work (Q1), as well as the tendon from where tenocytes were derived (Q3) and the statistical analysis performed (Q13), being attributed to all studies the highest score (2.0) in these items. The majority of the studies compared their results with other literature and, for this reason, Q9 was also one of the highest rated items. Half of the studies satisfactorily reported (rated 2.0) the number of samples utilized or species^17,27,28^ (Q2), the frequency of exposure^16,26,27^ (Q10), and the control and exposure groups^16,26,29^ (Q12). In general, studies clearly stated their outcomes (Q7) and the results were easily interpreted (Q6). The ethical committee approval was satisfactorily reported in 3 studies^16,17,28^ (Q11).

### Risk of bias assessment

**Figure 2:**
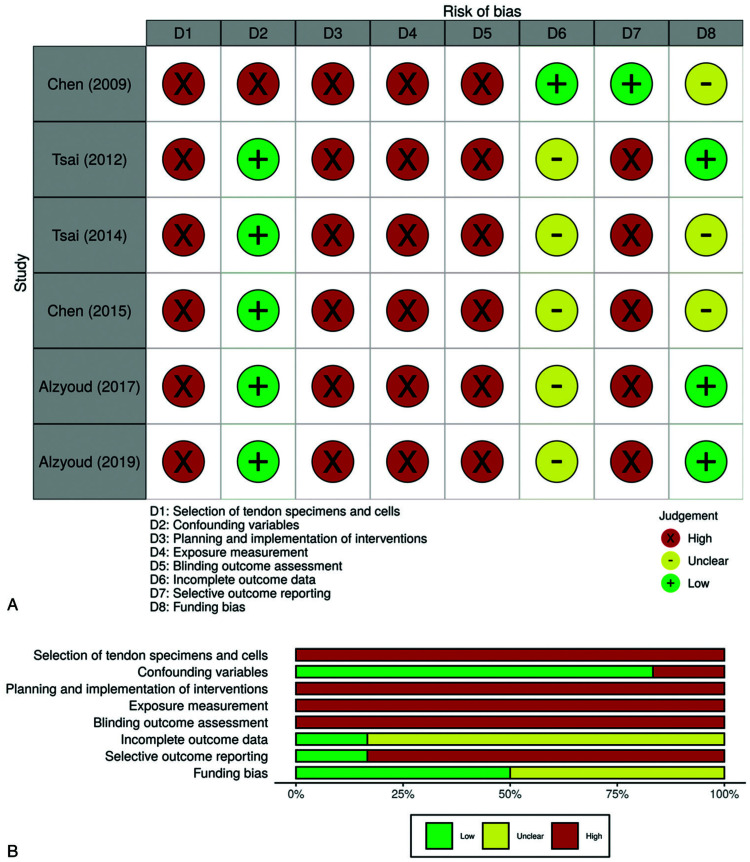
(A) Traffic light and (B) weighted summary and plots for the risk of bias assessment.

There was high selection bias due to inadequate selection of tendon specimens and cells in all 6 studies (Fig. 2). The reporting of the anatomical location from where tendons were excised, the weight, age and number of animals, and the randomization process were imprecisely reported. None of the studies confirmed cell surface markers. Half of them^16,17,28^ assumed that the isolated cells were tenocytes by analyzing their shape and growth rate. The other half^26,27,29^ did not present results for cell confirmation. Only 1 study (16.7%)^29^ presented high risk of selection bias due to confounding as it included uncontrolled confounding variables. However, cell viability and count, and culture medium volume were inconsistently reported in all studies and therefore the influence of these 2 potentially confounding variables could not be assessed. All studies were judged as high risk of performance and detection bias due to inadequate planning and implementation of interventions, exposure measurement, and blinding outcome assessment. Most studies (83.3%)^16,17,26−28^ showed unclear risk of attrition bias because not enough data were provided to verify if there was data loss, but only 1 study (16.7%)^29^ had low risk of reporting bias. Three studies (50.0%)^16,17,29^ were classified as unclear risk of bias for the funding bias domain due to the inability to ascertain if the authors had any conflict of interest regarding the results of their published study.

### Species, number of samples, and tendon

Half of the animal species were rats.^16,17,28^ Other species included porcine,^29^ bovine,^26^ and sheep.^27^ At least one third of studies included more than 10 samples,^17,28^ mostly from the Achilles tendon^16,17,27−29^ (83.3%) and collected in laboratorial settings^16,17,28^ (50.0%). Appendix C available in Supplementary Digital Content, http://links.lww.com/PBJ/A11 and Table 2 present the summary of these variables.

**Table 2:** Overview of the data extraction criteria for each study included in the present systematic review.

First author, year	Species (n)	Tendon	Light source stimulation parameters	Experimental conditions
Chen (2009)^29^	Porcine (n = NI)	Achilles	Type: GaAs laser and GaAs, In, P laser	DCL (cm): NI
			Operation mode: pulsed (frequency: 50 Hz)	CP: NI
			Number of actuators: NI	NS: 1
			AP: laser beam emitted perpendicularly to culture plates	Note: porcine ankle sections were purchased from a local wholesale meat supplier
			λ (nm): 820 (GaAs laser); 635 (GaAs, In, P laser)	
			RA (cm^2^): NI	
			P (mW): 40	
			PD (mW/cm^2^): NI	
			E (J): NI	
			ED (J/cm^2^): G1 = 0.0; G2 = 1.0; G3 = 2.0; G4 = 3.0	
			IT (s): G1 = 0; G2 = 540; G3 = 1080; G4 = 1620	
Tsai (2012)^28^	Sprague-Dawley	Achilles	Type: laser	DCL (cm): 30
	rat (n = 16)		Operation mode: continuous	CP: 2-4
			Number of actuators: array of 20 actuators (according to the manufacturer information)	NS: 1
				Note: tendon excised from rats weighing 200–250g
			AP: laser beam emitted perpendicularly to culture plates	
			λ (nm): 660	
			RA (cm^2^): 314	
			P (mW): 50	
			PD (mW/cm^2^): NI	
			E (J): NI	
			ED (J/cm^2^): G1 = 0.0; G2 = 1.0; G3 = 1.5; G4 = 2.0	
			IT (s): G1 = 0; G2 = 312; G3 = 468; G4 = 624	
Tsai (2014)^17^	Sprague-Dawley rat (n = 16)	Achilles	Type: laser	DCL (cm): 30
			Operation mode: continuous	CP: 2 and 4
			Number of actuators: array of 20 actuators (according to the manufacturer information)	NS: 1
				Note: tendons were excised from rats weighting 200–250g
			AP: laser beam emitted perpendicularly to culture plates	
			λ (nm): 660	
			RA (cm^2^): 314	
			P (mW): 50	
			PD (mW/cm^2^): NI	
			E (J): NI	
			ED (J/cm^2^): G1 =0.0; G2=1.0; G3=1.5; G4=2.0; G5=2.5	
			IT (s): G1 = 0; G2 = 312; G3 = 468; G4 = 624; G5 = 780	
Chen (2015)^16^	Sprague-Dawley rat (n=NI)	Achilles	Type: infrared GaAs diode laser	DCL (cm): NI
			Operation mode: pulsed (frequency range: 5000-7000 Hz, pulse duration: 200ns)	CP: 3–5
				NS: 1
			Number of actuators: 1	Note: tendons were excised from rats weighting 200–250g (laboratory)
			AP: NI	
			λ (nm): 904	
			RA (cm^2^): 0.07 (spot size)	
			P (mW): 2.4 (average) and 27,000 (maximum)	
			PD (mW/cm^2^): NI	
			E (J): NI	
			ED (J/cm^2^): G1 = 0.0; G2 = 0.5; G3 = 1.0; G4 = 2.0; G5 = 4.0	
			IT (s): NI	
Alzyoud (2017)^26^	Bovine (n=NI)	Deep digital flexor tendon	Type: intense pulsed light (IPL)	DCL (cm): NI
			Operation mode: pulsed (frequency: average or 0.25 Hz, pulse duration: 10-110ms, single pulse)	CP: 3
				NS: 2 with a 48h interval
			Number of actuators: NI	Note: cells isolated from adult bovine.
			AP: directly to the primary cell monolayer through the under surface of the culture plates	G1 denotes all control groups.
			λ (nm): 530-1100	
			RA (cm^2^): 8.9 (spot size on tissue)	
			P (mW): NI	
			PD (mW/cm^2^): NI	
			E (J): NI	
			ED (J/cm^2^): G2 = 10.0 (7.3); G3 = 15.0 (10.8); G4 = 20.0 (15.9)^*^	
			IT (s): NI	
Alzyoud (2019)^27^	Sheep (n =10)	Achilles	Type: light-emitting diode (LED)	DCL (cm): NI
			Operation mode: NI	CP: 3
			Number of actuators: LED array (number not specified)	NS: 1 or 2
				AP: top surface of a black 96-well culture plate	(optimization); 1 for experimental design
				λ (nm): 625 and 850	G1: control
				RA (cm^2^): 15.0 (spot area)	G2: LED
				P (mW): 1200	G3: PRP
				PD (mW/cm^2^): NI	G4: LED+PRP
				E (J): NI	Note: cells isolated from adult sheep
				ED (J/cm^2^): G1: 0.0; G2.1: 4.0; G2.2: 8.0; G2.3: 20.0	(slaughterhouse).
				IT (s): G1: 0; G2.1: 1080; G2.2 and G2.3: NI	

*λ* = wavelength (nm), AP = actuator positioning, CP = cell passage, DCL = distance from cells to light source, E = energy (J), ED = energy density (J/cm^2^), G1 = group 1 or control group, G2.0 = subgroup 0 of group 2 or control group, G2.1 = subgroup 1 of group 2, G2.2 = subgroup 2 of group 2, G2.3 = subgroup 3 of group 2, G2 = group 2, G3 = group 3, G4 = group 4, G5 = group 5, IPL = intense pulsed light, IT = irradiation time (s, seconds), LED = light-emitting diode, n = number of samples, NI = no information, NS = number of sessions, P = power (mW), PD = power density (mW/cm^2^), RA = reported area (cm^2^). The authors applied the IPL device settings with energy densities of 10, 15, and 20 J/cm^2^. However, the actual energy density delivered was determined to be 7.3,10.8, and 15.9 J/cm^2^, respectively. G1 regards to control groups utilized.

### Light parameters and experimental conditions

More than half of studies (66.7%) used lasers as the light source.^16,17,28,29^ Other light sources included LEDs^27^ and intense pulsed light (IPL).^26^ The range of wavelengths utilized by the 6 studies varied greatly, with the lowest and highest wavelengths being 530 and 1100 nm, respectively. These upper and lower bounds pertain to the same study.^26^ Three studies^26,27,29^ analyzed more than 1 wavelength.

Half of the studies positioned the light source perpendicularly to the culture plates.^17,28,29^ One study applied the light treatment directly to the primary cell monolayer through the under surface of the culture plate^26^ and another irradiated the top surface of the plate.^27^ The other study^16^ did not mention the light stimulus application positioning. Two studies (33.3%)^17,28^ irradiated the culture plate from above at a distance of 30cm. The number of cell passages was heterogenous across studies.

Most of the studies (83.3%) employed only 1 light treatment, except from 1 study. Alzyoud et al^26^ applied 2 sets of treatments with a 48 hours interval between them (2 sessions). For all studies, the time during which tenocytes were irradiated varied between 14.6 seconds and 1620.0 seconds.

### Analysis of results

Considering the analysis of results at the protein level, both the Western Blot and enzyme-linked immunosorbent assay were the most commonly utilized techniques (22.2%). At the mRNA and DNA levels, the reverse transcript polymerase-chain reaction and 4′,6-Diamidino-2-Phenylindole were the only techniques used, respectively (Appendix D available in Supplementary Digital Content, http://links.lww.com/PBJ/A11). Cell proliferation, mRNA expression of target proteins, cell migration, and cellular viability were the most reported outcomes.

### Main findings

Laser interventions significantly improved cell proliferation as compared to control groups. According to 2 studies, cell proliferation peaked at 2.0J/cm^2^.^17,29^ In turn, laser energy densities of 3.0J/cm^2^ reduced cell proliferation.^29^ Laser significantly increased tenocyte viability with 0.5 and 1.0J/cm^2^ in comparison to control group.^16^ The study using IPL stimulation observed a higher viability in cells treated with 15.9J/cm^2^ compared to the control group and the other IPL-treated groups.^26^ LED energy density of 20.0J/cm^2^ significantly decreases tenocyte viability, but not when using 4.0 J/cm^2^.^27^ Tenocytes treated with LED every other day presented significantly higher viability than the ones treated daily.^27^ While 15.9J/cm^2^ IPL and 4.0J/cm^2^ LED stimulation did not significantly affect tenocyte migration,^26,27^ laser stimulation with 1.0, 1.5, and 2.0 J/cm^2^ increased it.^28^

The mRNA expression of decorin, dynamin 2, type I collagen, proliferating cell nuclear antigen (PCNA), and transforming growth factor-β1 (TGF-β1) was significantly increased after laser interventions.^16,28,29^ Protein expression of PCNA and dynamin 2 was also up-regulated after laser treatment.^17,28^ No significant differences were found for mRNA expression of collagen type III and nitric oxide (NO) production during 1.0J/cm^2^ laser treatment,^16^ but 1 study found that laser dose-dependent significantly increased NO concentration.^17^ A 1.0 J/cm^2^ energy density resulted in significantly higher collagen synthesis and concentration of TGF-β1 in culture media.^16^

ATP production and intracellular calcium concentration increased after 1.0 J/cm^2^ laser treatment as compared to the control group.^16^ The optimal stimulation conditions for LED^27^ were 4.0 J/cm^2^ applied every other day and for IPL^26^ were 15.9 J/ cm^2^, phenol-red containing culture media supplemented with 10% fetal bovine serum.

Table 3 presents the techniques utilized to assess results and the main findings of all studies.

**Table 3:** Overview of the statistically significant main results.

First author, year	Techniques for analysis of results	Main findings (statistically significant)
Chen (2009)^29^	MTT	↑ cell proliferation in G2 (13% ± 0.8%), G3 (30% ± 0.4%), and G4 (12% ± 0.6%), comparing to G1.
	RT-PCR	Higher laser intensity (3.0J/cm^2^) ↑ higher cell proliferation. Most effective: G3
		↑ mRNA expression of decorin and type I collagen in laser-treated groups compared to the control group.
Tsai (2012)^28^	iv-WHM	↑ migration of tenocytes across the wound border with laser treatment.
	TFMA	↑ cell migration through the filters dose-dependently in laser-treated groups: G2 (118.8 ± 4.6%), G3 (133.7 ± 9.0%), and G4 (156.5 ± 11.1%), comparing to G1. Statistically significant differences between G1 and G2, G2 and G3, and G3 and G4.
	Quantitative RT-PCR	↑ mRNA expression of dynamin 2 after laser treatment dose-dependently: G2 (1.02 ± 0.02), G3 (1.14 ± 0.02), and G4 (1.35 ± 0.01). Statistically significant results between G2 and G3 and G3 and G4.
	WB	↑ dynamin 2 protein expression with laser dose-dependently.
	IFS	↑ cellular protein expression of dynamin 2 in tenocytes cytoplasm in laser-treated group comparing to control group.
		The migration of tenocytes treated with 2.0J/cm^2^ was significantly suppressed by dynasore treatment.
Tsai (2014)^17^	MTT	↑ number of viable cells by laser treatment in a dose-dependent manner: G2 (102.2 ± 2.5%), G3 (103.6 ± 3.0%), G4 (112.8 ± 3.3%), and G5 (109.6 ± 8.2%), comparing to G1. Significant results between G1 and G4 and G1 and G5.
	ICC	↑ tenocyte proliferation indicated by the higher positively stained with fluorescent green tenocytes in the laser groups compared to control. The percentage of Ki-67 positive tenocytes increased dose- dependently after laser treatment: G1: 53.6 ± 9.1%, G2: 66.4 ± 10.0%, and G4: 76.4 ± 0.7%.
	ELISA	↑ NO secretion and protein expression of PCNA and cyclins E, A and B1 after laser treatment compared to control.
	WB	Laser with 2.0J/cm^2^ resulted in the most significant cell proliferation, NO secretion and PCNA protein expression.
Chen (2015)^16^	MTT	↑ OD value for laser-treated group at 24 h (0.068 ± 0.007, 0.073 ± 0.011, 0.065 ± 0.008, and 0.064 ± 0.004 for G2, G3, G4, and G5, respectively) and 48h (0.103 ± 0.006, 0.106 ± 0.012, 0.104 ± 0.012, and 0.100 ± 0.011 for G2, G3, G4, and G5, respectively) than the control group at 24 h (0.06 ± 0.003) and 48 h (0.093 ± 0.011) cell viability for G2 and G3 at 24 h and 48 h in comparison with G1.
	SCA	For 1.0J/cm^2^:
	ELISA	↑ collagen synthesis in culture media in comparison with the control group.
	ATP-CA	↑ concentration of TGF-β1 in the culture medium at 12 h, 48h, and 72 h compared with the control group.
	Greiss-R	↑ ATP production (at 15min, 30min, and 4h) and intracellular Ca^2+^ concentration after laser treatment (15 and 30min).
	Fluo-3AM	↑ mRNA expression of PCNA (after 24h), type I collagen (after 24h), and TGF-β1 (after 72 h) after laser treatment verified by quantitative PCR analysis.
	Quantitative RT-PCR	
Alzyoud (2017)^26^	AB	Cell viability increased with the increase in FBS concentration (0%, 5%, and 10%) and different culture media (PRC-CM or without phenol red).
	SA	↑ cell viability with 2 IPL treatments of 15.9J/cm^2^, PRC-CM over 96h of culture period comparing to the other groups.
	Immunolabelling (live/dead staining)	↑ cell viability with 15.9 J/cm^2^ when compared to control group, 7.3 J/cm^2^ and 10.8 J/cm^2^.
		Optimal stimulation conditions: 15.9 J/cm^2^, PRC-CM supplemented with 10% FBS.
Alzyoud (2019)^27^	TBM	LED optimal conditions: 4.0 J/cm^2^ applied every other day (48 h period).
	AB	↑ cell proliferation and viability with 10% FBS.
	DAPI	↓ viability in cells treated with 20.0J/cm^2^ compared to control.
	SA	Cell viability in every other day treatment period was significantly higher than daily treatment period.

AB = Alamar Blue assay, ATP = adenosine triphosphate, ATP-CA = ATP colorimetric assay, Ca^2+^ = calcium ion, CM = culture media, DAPI = 4′,6-Diamidino-2-Phenylindole, ELISA = enzyme-linked immunosorbent assay, FBS = fetal bovine serum, Greiss-R = Greiss reaction, G1 = group 1 or control group, G2 = group 2, G3 = group 3, G4 = group 4, G5 = group 5, ICC = immunocytochemistry, IFS = immunofluorescence staining, IPL = intense pulsed light, iv-WHM = in vitro Wound Healing Model, LED = light-emitting diode, mRNA = messenger ribonucleic acid, MTT = (3-[4,5-dimethylthiazol-2-yl]-2,5-diphenyltetrazoliumbromide assay, NO = nitric oxide, OD = optical density, PCNA = proliferating cell nuclear antigen, PRC-CM = phenol red-containing culture media, RT-PCR = reverse transcript polymerase chain reaction, SA = scratch assay, SCA = Sircol Collagen Assay, TBM = Trypan Blue Method, TFMA = Transwell Filter Migration Assay, TGF-β1 = transforming growth factor-β1, WB = Western Blot analysis.

## Discussion

The present systematic review provides an overview of the methodologies, stimulation parameters, and main outcomes of available in vitro studies after light stimulation on tenocytes. The gathered information is a valuable tool for other researchers to reproduce, compare, adapt, and improve their experimental procedures according to the particular needs of their research projects. Even though not being directly extrapolated to in vivo studies, the in vitro results found in this systematic review may be a promising starting point for the investigation of the application of light sources to both animals and humans.

Primary cell lines are constituted by cells directly isolated from an animal tissue, being grown in plastic dishes containing fundamental nutrients and serum to provide them with optimal cell growth, division, and survival conditions.^30^ All 6 studies analyzed in this review used primary cell lines, since the cells utilized in their experiments were derived from animal tendons. Cells were derived from 4 different animal species, namely porcine, Sprague-Dawley rats, bovine, and sheep. By utilizing different animal species, differences inevitably arise. These 4 animal species have distinctive anatomies, their tendons are subjected to different levels of mechanical tension, they perform different movements, their nutrition is also different, and they have different modes of performing the same activities. Their tendons, and consequently the tenocytes constituting those tendons, are subjected to different environmental conditions and, thus, a comparison between them is restricted. The testing of light stimulation on human tenocytes was not available.

The animal samples were acquired from different sources, namely local meat supplier (porcine), laboratory (Sprague-Dawley rats), and slaughterhouse (sheep). The place where bovine samples were acquired from was not mentioned. By acquiring samples in a local meat supplier, where animals are already dead for an undetermined period of time upon purchase and utilization for experiments in laboratory, is different from acquiring samples from a local slaughterhouse. This is even different from acquiring animal samples from an animal laboratory, where animals are euthanized, and their cells are utilized for experiments at a time pre-determined by researchers, enabling more control of the experimental conditions. Future studies should make efforts towards using tenocytes under restrict experimental conditions where researchers are able to control the animals’ previous activity and living conditions.

Cell passaging is a process involving transferring a small number of cells into a new container, which can be a culture plate well, dish, vessel, or flask. This is a very important aspect of the study, as higher passage number may induce cellular alterations in the growth rates, morphology, and response to stimuli, amongst others, when compared to lower cell passage number.^31−35^ The tenocyte passage during light stimulation experiments is an important experimental condition to be considered. The cell passage number varied between 2 and 5, with 3 being the most common (33.3%). One study^29^ did not mention the cell passage. To which extent this variation has affected the results is not possible to quantify. One study determined however that third and seventh cell passage did not significantly affect the rate of cell migration.^26^

The heterogeneity in the above-discussed experimental conditions may induce different pre-stimulation conditions in samples, which may result in different experimental conditions in cell culture, and thus different results. These disparities encountered in the methodologies employed by the studies preclude a precise comparison amongst studies regarding their main results and conclusions.

Wavelength, power, irradiation time, beam area at the skin or culture surface, pulse parameters, anatomical location, number of treatments, and interval between treatments are the beam and actuation parameters that must be reported in studies investigating the effect of light sources, as defined by Jenkins and Carroll.^36^

Dose parameters, which include energy and energy density, must also be detailed. By correctly and accurately reporting these parameters, researchers enable the reproducibility of their scientific studies, as well as significantly increase the scientific information presented in published works, enhancing their value and applicabil-ity.^36^ Amongst these parameters, we evaluated wavelength, power, irradiation time, area, pulse parameters, number of treatments, interval between treatments, energy, and energy density. The anatomical location was not relevant to our review since in vitro studies are being evaluated and light stimulation is being applied to cell cultures. Pulse parameters (pulse on and off duration, and duty cycle) were inadequately reported by the 6 included studies. All 6 studies reported the wavelength, energy density, and number of sessions or treatments employed in their experimental works. The area, power and irradiation time were reported in some studies. Even though most studies reported most of these parameters, no study reported the entire set of parameters recommended by Jenkins and Carroll.^36^ Future studies should make an effort to standardize the reporting of irradiation parameters to enable reproducibility and precise research on this scientific area.

The biochemical responses of tenocytes to light stimulation were also evaluated. Regarding the use of laser, an energy density of 2.0J/cm^2^ was the most effective value employed amongst studies. The laser actuation in these conditions promoted higher cell proliferation and viability,^17,29^ higher cell migration, higher mRNA, and protein expression of dynamin 2,^28^ as well as higher NO secretion and PCNA protein expression.^17^ Chen et al^16^ found higher mRNA expression of PCNA, TGF-β1, and type I collagen following a 1.0J/cm^2^ stimulation. Higher cell viability was promoted at 0.5 and 1.0 J/cm^2^, while 1.0 J/cm^2^ stimulation promoted higher ATP production and intracellular Ca^2+^ concentration.^16^ Nevertheless, it is important to highlight that even though similar types of light source were used, other light stimulation parameters differed amongst studies. Chen et al^29^ and Tsai et al^28^ applied 40 and 50 mW power laser sources, respectively. These differences in light parameters preclude more complete comparisons and conclusions between studies. Chen et al^29^ reported that high laser intensities (3.0 J/cm^2^) would not promote higher cell proliferation, but 2 authors concluded differently. Alzyoud et al^26^ determined that the optimal stimulation conditions were achieved with an IPL energy density of 15.9J/cm^2^, which promoted higher cell viability. The same author^27^ determined that the optimal LED conditions for actuation was a 4.0 J/cm^2^ energy density, promoting higher cell viability, migration, and proliferation. However, as previously referred, the light sources are different and, consequently, the reported outcomes cannot be further compared.

The methodological quality of the 6 studies included in this systematic review of literature should also be addressed. The major concerns identified were related to the poor description of the experimental setup, equipment and evaluation procedures, identification of study’s limitations, and incomplete reporting of light parameters. The lack of proper and standardized reporting of these parameters hinders comparisons between results and makes it difficult to draw out conclusions amongst studies, precluding the direct comparison between studies and limiting the strength of the recommendations that can be made. For this reason, we suggest the report of the parameters presented in the summary table developed in our systematic review, accompanied by the checklist provided by Jenkins and Carroll.^36^ This strategy should be implemented in future studies to avoid inconsistent reporting of these parameters and enable a reliable and precise comparison amongst studies, as well as the reproducibility and applicability of their results. There was also high risk of bias in most of bias domains (selection bias, performance bias, detection bias, and reporting bias), which decreases the confidence of the results of the included studies.

Some limitations of this systematic review are important to be mentioned. This review only included studies analyzing the effect of a light source on tenocytes in vitro. However, when the study utilized a light source combined with other technique, such as platelet rich plasma,^27^ the study was also considered but we only included the results relevant to our context. Studies utilizing animals in vivo to perform experiments and clinical trials, were also not considered for analysis. This led to the exclusion of several studies that, although reporting important aspects, did not fulfil the established eligibility criteria.

## Conclusion

The application of light therapy to tenocytes resulted in higher proliferation, viability, mRNA and protein expression of target biochemical outcomes, and migration. However, there is a high degree of heterogenicity in reporting methodologies employed in the experimental setup and a lack of standardized methods in reporting light stimulation parameters. This hinders more definitive and precise conclusions. The absence of reporting of studies’ limitations is also a major pitfall as it hampers the identification of current limitations and suggestion of future directions. These shortcomings should be carefully addressed in future research carried out in this scientific domain, aiming to clearly and systematically report the experimental parameters discussed in this review, including light and experimental setup parameters.
